# Detection of Genetic Patterns in Endangered Marine Species Is Affected by Small Sample Sizes

**DOI:** 10.3390/ani12202763

**Published:** 2022-10-14

**Authors:** Piero Cossu, Laura Mura, Gian Luca Dedola, Tiziana Lai, Daria Sanna, Fabio Scarpa, Ilenia Azzena, Nicola Fois, Marco Casu

**Affiliations:** 1 Department of Sciences for Nature and Environmental Resources, University of Sassari, 07100 Sassari, Italy; 2 Department of Veterinary Medicine, University of Sassari, 07100 Sassari, Italy; 3 Dipartimento per la Ricerca nelle Produzioni Animali, Agris Sardegna, 07040 Olmedo, Italy; 4 Department of Biomedical Sciences, University of Sassari, 07100 Sassari, Italy

**Keywords:** conservation genetics, effective population size, microsatellite markers, genetic monitoring, marine invertebrates, gene flow

## Abstract

**Simple Summary:**

Knowledge of genetic diversity is crucial to improve conservation plans for endangered species and ensure their long-term persistence. However, it may be difficult to accurately estimate genetic diversity when it is unfeasible to obtain sufficiently large sample sizes, which is often the case for marine endangered species. Here, we use *Patella ferruginea* as a model to investigate how small sample sizes affect genetic diversity estimates. We show that small sample sizes do not always hamper the detection of genetic patterns in endangered marine species, even though caution is needed when genetic divergence among populations is weak.

**Abstract:**

Knowledge of Genetic diversity and its spatial distribution is crucial to improve conservation plans for endangered species. Genetic tools help ensure species’ long-term persistence by unraveling connectivity patterns and evolutionary trajectories of populations. Here, microsatellite genotypes of individuals from populations of *Patella ferruginea* are used to assess the effect of sample size on metrics of within-and between-population genetic diversity by combining empirical and simulated data. Within-population metrics are slightly to moderately affected by small sample size, albeit the magnitude of the bias is proportional to the effective population size and gene flow. The power of detecting genetic differentiation among populations increases with sample size, albeit the gain of increasing the number of sampled individuals tends to be negligible between 30 and 50. Our results line up with those of previous studies and highlight that small sample sizes are not always a hindrance to investigating genetic patterns in endangered marine species. Caution is needed in interpreting genetic patterns based on small sample sizes when the observed genetic differentiation is weak. This study also highlights the importance of carrying out genetic monitoring in seemingly well-preserved but potentially isolated populations.

## 1. Introduction

The amount of genetic diversity within populations, its spatial distribution, and population connectivity have been recognized as important components of reserve design [1]. Indeed, preserving genetic diversity and gene flow among populations ensures the maintenance of species’ adaptive and evolutionary potential and is thus crucial to withstand the effects of anthropogenic pressures and environmental change on species’ long-term persistence and survival [2]. Therefore, genetic tools are increasingly embedded into conservation framework to help fulfill goals such as: assessing the effectiveness of protected areas and their networks to ensure population connectivity, designing conservation plans, site selection, and prioritizing conservation actions for threatened species [3,4,5]. Genetic approaches have been particularly useful in marine organisms when other methods, such as those that, for instance, directly track dispersal, are unfeasible [6].

Many coastal marine invertebrates are a noteworthy case in point as they are broadcast spawners with a sedentary lifestyle, which rely on larval stages for recruitment and dispersal. Thus, population connectivity and the spatial distribution of genetic diversity will depend on the interplay between species-specific life-history traits, nearshore oceanography, coastal topography, habitat availability and selection [7,8,9,10,11]. These factors may result in complex patterns of genetic variation over time and space [6,9,12,13]. Conservation practitioners must account for spatio-temporal genetic variation and thus genetic monitoring is needed to help tailor suitable conservation strategies to such species [14]. However, when dealing with endangered or rare species. obtaining adequate sample sizes may be an issue implying statistical, biological, and ethical concerns [15]. Both simulated [16,17] and empirical data based mainly on terrestrial species [18,19] have been used to investigate the sample size that is needed to obtain precise and accurate genetic metrics, whereas little is known about marine species as we are aware.

Here, we focus on assessing the minimum sample size required to achieve reliable estimates of such genetic metrics in *Patella ferruginea* Gmelin, 1791 (Mollusca: Gastropoda) using the available set of microsatellite markers [20]. As primary grazers, limpets play a key role in shaping the structure and functioning of rocky shore communities [21,22], as well as in influencing ecosystem stability [23]. As with other shellfish, limpets have faced a long history of human exploitation, being used either as food or bait for fishing [24,25]. Overharvesting resulted in the collapse of several populations and led to ecological changes in the structure of intertidal communities, underpinning further ecosystem-level impacts [22,26].

*Patella ferruginea*, which is endemic to the Mediterranean Sea, fits this picture and is listed among the most threatened marine macro-invertebrates of the Mediterranean (European Council Directive 92/43/EEC on the Conservation of Natural Habitat of Wild Fauna and Flora, 1992). Although the ‘strict protection’ status, the species is still subject to poaching and collection by fishermen as well as unaware tourists [27]. The species was widespread and abundant throughout the Pleistocene and started to decline during the Neolithic, as indicated by shell deposits [28]. The decline was gradual until the end of the 19th century, whereupon the species underwent a most severe decline, mainly because of direct and indirect anthropogenic pressure, which led to the current distribution by the first half of the 20th century [29,30]. Nowadays, the distribution of *P. ferruginea* is fragmented and restricted to the north African shoreline, Spain, Sardinia, Corsica, and a few scattered sites along Italian coasts [31,32]. Overall, large populations have been reported so far only along north African coasts [33,34], whereas the remaining areas mostly host relict populations [31,35], with the noteworthy exception of two marine protected areas (MPAs) in Sardinia [36]. Therefore, habitat fragmentation and the conservation status of many populations raise concerns for the species’ persistence and survival in the short as well as the long term. Quite a few studies have investigated the ecology and life-history of *P. ferruginea* [30,33,37,38,39,40,41,42,43], some of which were aimed at favoring the reintroduction and/or restocking of natural populations [29,44,45]. By and large, the above studies, pointed out that the interplay between life-history traits, scarce density and habitat fragmentation might hinder recruitment, population connectivity and gene flow among populations.

Notwithstanding this issue, few studies have investigated patterns of genetic diversity in *P. ferruginea*. Both mitochondrial and nuclear markers have been used to analyze genetic patterns at local and basin scales, yielding partly contrasting results [28,36,46,47,48]. Whilst the former indicated basin-scale genetic homogeneity, Inter Simple Sequence Repeat markers (ISSR) detected genetically divergent populations with a clear north-south spatial subdivision across the Sardinian channel and within the Sardinian–Corsican region [28]. Moreover, comparing spatial genetic patterns of *P. ferruginea* and *P. ulyssiponensis*, the former had a lower potential for dispersal than the latter [49]. Fine-scale genetic patterns based on microsatellite markers further shed light on the species’ dispersal capabilities, which ranged between 1 and 15 km, albeit most dispersal events likely occurred within a range of 2 km [36]. This study also highlighted that: (1) spatial genetic structure and dispersal likely depended on the interplay between biotic and abiotic factors; (2) levels of genetic diversity and effective population sizes were comparable to those of other marine invertebrates.

The present study aimed at laying the ground for the genetic monitoring of *P. ferruginea*, which is crucial to assess the status of conservation and health of populations as well as the spatial genetic patterns over ecological time scales. Within this framework, it is paramount to investigate the power of assessing genetic diversity and its spatial distribution when the sample size per population is small, which is often the case with endangered and/or rare species. To reach this goal, empirical data were used as an illustrative example of the genetic patterns resulting from small sample sizes, and simulations were carried out to assess the minimum sample size required to obtain reliable genetic information to fulfill conservation purposes.

## 2. Materials and Methods

### 2.1. Sampling and DNA Extraction

**Figure 1:**
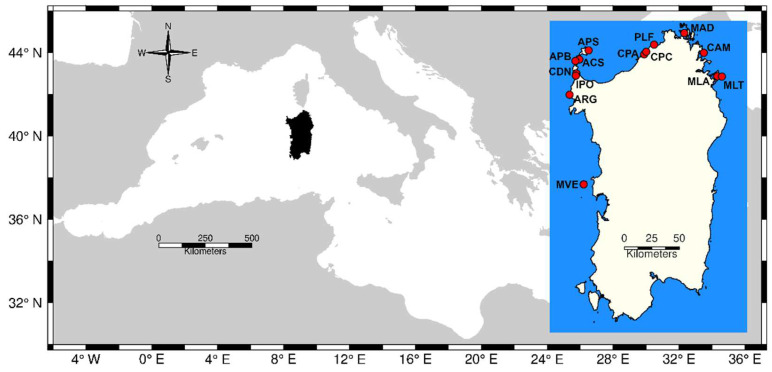
Location of sampling sites (red circles in the inset) in Sardinia Island (highlighted in black). APS = Punta Sabina, Asinara island; ACS = Cala Sant’Andrea, Asinara island; APB = Pedra Bianca, Asinara island; CDN = Coscia di Donna; IPO = Isola dei Porri; ARG = Argentiera; MVE = Mal di Ventre island; CPA = Costa Paradiso site A; CPC = Costa Paradiso site C; PLF = Punta Li Francesi; MAD = La Maddalena island; CAM = Le Camere island; MLA = Molara island; MLT = Molarotto island.

To develop and test the multiplex, old genomic DNA obtained from previous studies [28,50], which was stored at −20 °C, was used. Overall, 191 samples of genomic DNA from 29 localities (Table S1) were used to set up the multiplex amplification protocols. However, only localities whose sample size exceeded the threshold of five individuals were used for further analyses (Figure 1).

### 2.2. PCR Protocols

Using the singleplex PCR (Polymerase Chain Reaction) protocols as a starting point and the information on the allele size range of each locus outlined in a previous study [36], microsatellite loci were grouped into two multiplex reactions: Pf-D11A, Pf-G1M, Pf-31IB2 and Pf-31IB1 in Multiplex A (MpxA); Pf-31AH8, Pf-C10, Pf-G6A and Pf-31IF2 in multiplex B (MpxB). However, Pf-31IF2 was removed from MpxB as it performed poorly under multiplex PCR conditions. This locus was thus run as a singleplex reaction. Forward primers were labeled on the 5′ region with the fluorescent tags: VIC, 6-Fam and NED (Table 1). The PCR protocol was optimized until clear peaks for each locus were obtained in the ABI PRISM 3130xl Genetic Analyzer (Applied Biosystems). The optimization process mainly consisted in adjusting the concentration of each primer (Table 1) and annealing temperature, which was raised from 48 to 54 °C (see Table S2 for details on the protocol outline). Microsatellite alleles were scored using GENEMAPPER 4.0 (Applied Biosystems).

### 2.3. Statistical Analysis

Tests on departure from Hardy-Weinberg proportions, linkage disequilibrium, genotyping errors (e.g., presence of null alleles), potential outliers for selection were not carried out in the present study as the loci were the same used in a previous study [36]. In brief, only one locus, Pf-G6A, showed a significant heterozygote deficit, which could likely be attributed to the presence of null alleles. Nevertheless, the frequency of null alleles was below the threshold above which the bias might affect genetic analyses and thus the locus was retained for downstream analyses.

Within the R 4.0.2 statistical environment [51], the package DiveRsity [52] was used to compute within- and between-population summary statistics. The function divBasic() was used to estimate genetic diversity within each population by computing the number of alleles (*N*_A_), Allelic richness (*A*_R_), Expected and observed heterozygosity (*H*_E_ and *H*_O_), as well as the inbreeding coefficient (*F*_IS_). Genetic differentiation among populations was estimated using the function diffCalc() to compute Weir and Cockerham’s *F*_ST_ estimator *θ* [53] and Hedrick’s *G*’_ST_ [54]. A bootstrap procedure with 10,000 replicates was used to estimate the 95% confidence intervals of *θ* and *G*’_ST_. A Fisher’s exact test was further used to assess population differentiation among sites: the function chiCalc() was used to test for genetic heterogeneity by carrying out 100,000 Montecarlo simulations. The joint probability of population differentiation was computed by combining single locus probabilities with Fisher’s method. Probability values at single loci were limited to *p* = 0.0001 to avoid a single locus having an overwhelming impact on the global probability [55]. For pairwise population comparisons, correction for multiple testing was carried out according to Narum [56]. Moreover, GenAlex 6.51b2 [57] was used to compute two further metrics of within-population genetic diversity: the unbiased expected heterozygosity (u*H*_E_), which accounts for finite sample size, and the effective number of alleles (*A*_E_).

The effect of sample size on within-population metrics of genetic diversity was investigated by simulating isolated populations that underwent bottlenecks of different intensities. Simulations were set up to mimic as close as possible the biological traits and the demographic history of *P. ferruginea* [30]. First, forward simulations allowing for overlapping generations were carried out using BottleSim 2.6 [58] to create in-silico populations with different effective population sizes (*N*_e_). Since BottleSim can use empirical genetic data, individual genotypes from Tavolara MPA [36] were used as input. Three scenarios were simulated, all of which assumed: constant population effective size during the bottleneck, monoecy with random mating without selfing, random starting age for all individuals (100% of overlap degree across generations), expected longevity of 20 years with sexual reproductive maturity set at two years. All scenarios started at year 0 with *N*_e_ = 500 individuals and differed for the intensity of the bottleneck: no population size reduction (*N*_e_ = 500 all over the entire simulation), moderate (five-fold) and severe (10-fold) population size reduction (*N*_e_ = 100 and 50 after year 1, respectively). Each scenario was run for 100 years and replicated 1000 times. The R-package PopGenKit 1.0 [59] was used to prepare the input file for BottleSim and to convert back output files from simulations into Genepop format (i.e., allele codes used by BottleSim were replaced by the original allele sizes).

Then, ten simulated datasets were randomly chosen from each scenario to assess the effect of sample size on allelic diversity: random sub-sampling was carried out using a customized version of Keenan’s R script: each dataset was resampled 100 times with replacement for each level of sampling intensity ranging from 4 to 48 individuals, and then diveRsity was used to compute the allelic richness for each locus in each resampled dataset. The mean and 95% confidence interval of allelic diversity were then computed over the resampled datasets for each sampling level.

To assess the effect of sample size on the power of detecting population differentiation, we used a framework similar to that adopted by a previous study [16]. Powsim 4.0 [60] was used to simulate scenarios in which genetic drift was the only evolutionary force driving genetic divergence among populations (no migration, selection nor mutation are accounted for). As a baseline for simulations, allele frequencies from a previous study [36] were used to simulate pseudo-datasets with the same number of populations (*N*), loci, alleles and sampling size (*S*) as the real dataset. Different levels of genetic differentiation (*F*_ST_ = 0.00–0.1) were simulated assuming constant population effective size (*N*_e_ = 1000) and varying the number of generations since divergence (*t* = 0, 2, 5, 10, 15, 20, 50, 102 generations). The statistical power was evaluated by computing the fraction of both *Χ*^2^ and Fisher’s exact tests that successfully detected population differentiation out of 1000 replicates for each simulation. Three further sets of simulations were carried out by increasing the sample size (*S* = 20, 30, 50 individuals). To account for different effective population sizes and some level of migration across demes, the connectivity module available in SpotG, an online simulation tool (http://www.congressgenetics.eu/simulatorhome.aspx, last accessed on 3 March 2021) was also used [61,62,63]. Genetic divergence was simulated among 10 populations (the maximum number of demes allowed by the online tool) under a symmetric finite island model. Since the module cannot use empirical allele frequencies as a baseline, 8 microsatellite loci with a mutation rate equal to 0.0005 were simulated. Different sample sizes (*S* = 10, 20, 30, 50), effective population sizes (*N*_e_ = 50, 100, 500, 1000) and migration rates (*m* = 0.001, 0.005, 0.01, 0.05, 0.1) were combined to obtain 60 distinct scenarios, each of which was replicated 1000 times. Overall power was computed as the proportion of times out of 1000 iterations in which genetic differentiation was successfully inferred. All data and R scripts used to generate the analyses are available on FigShare at https://doi.org/10.6084/m9.figshare.14611407.v1.

## 3. Results

The multiplex tool developed for *P. ferruginea* successfully amplified 173 out of 191 tested individuals. However, after keeping only those sites whose sample size exceeded five individuals, the final dataset consisted of 131 individuals, which were spread across 14 sites (Figure 1). Sample sizes ranged between 8 and 16 individuals per site with mean and variance of 9.36 and 4.71, respectively (Table S1). Overall, 84 alleles were scored across all loci and sites, with the number of alleles per site ranging from 35 (ACS, Cala Sabina, Asinara island) to 50 (CDN, Coscia di Donna) (Table S3).

### 3.1. Patterns of Genetic Diversity

Within population summary statistics for each site are reported in Table 2. Overall, all sites but MVE (Mal di Ventre island) displayed moderate levels of expected and observed heterozygosity as both metrics were below 0.70; the MVE population, which is located within an MPA, slightly exceed this threshold for observed heterozygosity (*H*_O_ = 0.73 ± 0.11). Moreover, albeit the expected heterozygosity was below (*H*_E_ = 0.65 ± 0.10), the unbiased heterozygosity reached the above threshold (u*H*_E_ = 0.70 ± 0.11). In contrast to MVE, which showed the highest values, another site from a MPA, Punta Sabina, Asinara island (APS), showed the lowest values for these metrics (*H*_E_ = 0.55 ± 0.10, u*H*_E_ = 0.58 ± 0.12, *H*_O_ = 0.53 ± 0.11). Most sites (9 out of 14) showed a slight excess of heterozygotes as evidenced by the small negative values of the inbreeding coefficient (*F*_IS_, Table 2). The largest values of heterozygote excess (*F*_IS_ > 0.10) were recorded at IPO (Isola dei Porri), MVE, PLF (Punta Li Francesi), and MAD (La Maddalena). MVE also showed the largest value of the effective number of alleles (*A*_E_ = 4.27 ± 0.71) but not for allelic richness: interestingly, an unprotected population located on the mainland, Coscia di Donna (CDN), showed the largest values for this metric (*A*_R_ = 4.64 ± 0.65). As well, a site from Asinara MPA (ACS, Cala Sant’Andrea) displayed the lowest values for both metrics (*A*_R_ = 3.72 ± 0.52 and *A*_E_ = 3.15 ± 0.50, respectively).

Populations included in the present study did not show significant genetic differentiation (Fisher’s exact test, *p* = 0.084). Both Weir and Cockerham’s *θ* and Hedrick’s *G*’_ST_ were small, showing 95% confidence intervals that spanned zero (*θ* = 0.0003, CI = −0.017–0.020; *G*’_ST_ = −0.0006, CI = −0.041–0.047). Although 21 and 34 out of 91 population pairs displayed pairwise values of genetic differentiation ≥ 0.01 for both *θ* and *G*’_ST_, respectively (Table 3), no population was genetically divergent from other populations after correcting for multiple testing (adjusted Fisher’s exact test, *p* > 0.05). Moreover, all pairwise comparisons displayed large confidence intervals that spanned zero for both metrics of genetic differentiation (Table S4).

### 3.2. Effect of Sample Size on Genetic Diversity within Populations

The change in genetic diversity over time is illustrated in Figure S1. Results showed that even an unbottlenecked population (*N*_e_ = 500) underwent a loss of genetic diversity. On average, about 18% of alleles were lost by genetic drift; conversely, expected and observed heterozygosity showed a negligible decrease as both metrics dropped to 99 and 98.5% of their starting value, respectively. The number of effective alleles decreased only by 5%, from 4.53 ± 1.07 to 4.29 ± 0.95. In contrast, the number of alleles dropped to 48 and 61% of the starting value when the population effective size underwent a five- and ten-fold reduction, respectively. Noteworthy, whilst a population with *N*_e_ = 50 lose three times more alleles than an unbottlenecked one (*N*_e_ = 500), the decrease of effective alleles was roughly seven times larger in the small (*A*_E_ = 2.89 ± 1.07) compared to the large population. Even bigger was the relative loss of *H*_E_ and *H*_O_ related to an unbottlenecked population, which dropped to 87 and 84% of their starting values, respectively (Figure S1).

**Figure 2:**
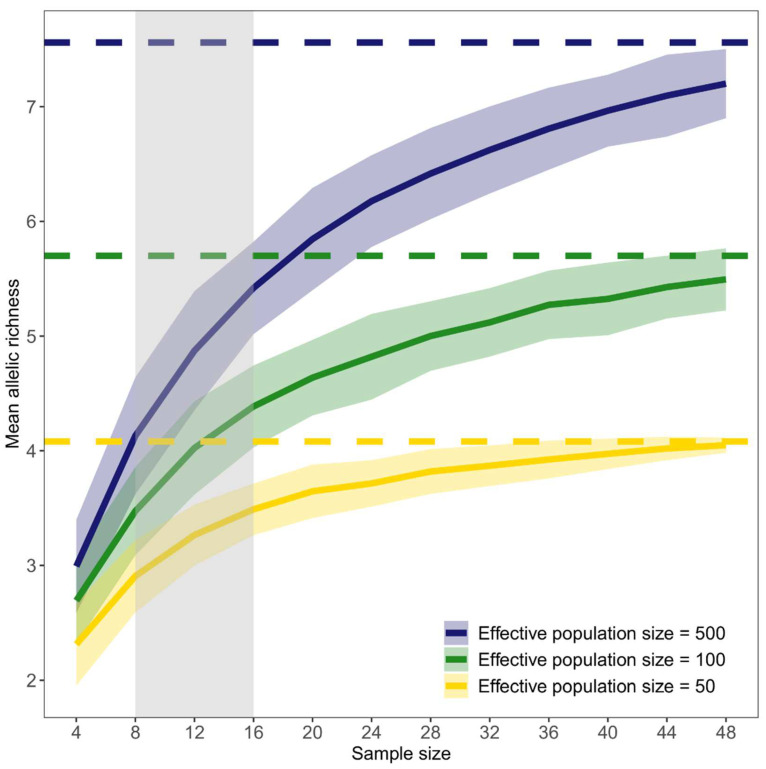
Effect of increasing sample size on genetic diversity for different effective population sizes. Solid lines represent the mean allelic richness and shaded areas around each line outline 95% confidence intervals, estimated on 100 resampled datasets for each sample size. Dashed lines represent mean allelic richness assessed over a sample size of 50 individuals. The gray-shaded area encompasses the sample size interval of the real data.

These differences affected the outcome of random subsampling, which outlined as allelic diversity (assessed as mean allelic richness over loci) increased at increasing sample sizes (*S*). The three randomly selected populations, which are depicted in Figure 2, highlighted that the sample size at which allelic diversity approached an asymptote (here the value at *S* = 50) was inversely proportional to the effective population size. For instance, in a population with a small effective size (*N*_e_ = 50), <10 individuals are sufficient to detect 71% of the allelic diversity that was assessed with S = 50 (*A*_R_ = 2.91 and 4.08, respectively). in a large population (*N*_e_ = 500), a similar proportion was obtained by sampling twice the individuals (*A*_R_ = 5.42 and 7.56 for *S* = 16 and 50, respectively). Figure S2, which depicted the curves of a further nine randomly selected populations for each of the three simulated scenarios, confirmed this trend, in which small sample sizes underestimated genetic diversity and the bias tended to be more severe as effective population size increased.

### 3.3. Effect of Sample Size on Genetic Differentiation

**Figure 3:**
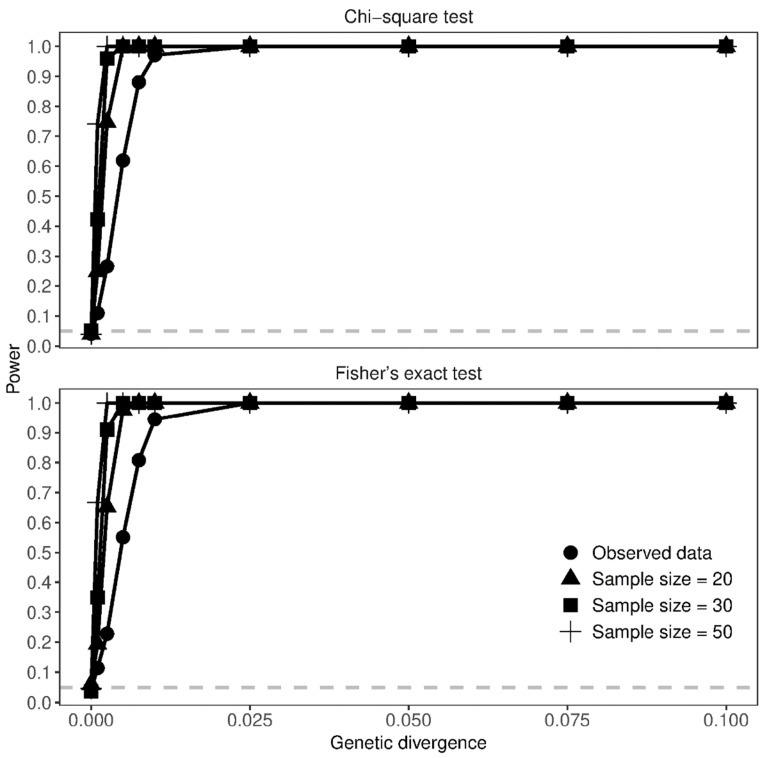
Sample size effect on the power of detecting population differentiation in isolated populations. Different *G*’_ST_ values (x-axis) were obtained by increasing the number of generations since the split of populations, whereas population effective size and migration were constant (*N*_e_ = 1000 and *m* = 0, respectively). The proportion of significant tests over 1000 simulated data for each scenario (y-axis) corresponds to *α*-error when *G*’_ST_ = 0 and to power otherwise. The gray-dashed line outlines the threshold of the expected *α*-error (*α* = 0.05).

Small sample sizes did not always preclude the chance of successfully detecting population differentiation even when genetic divergence was weak. Figure 3 showed that a sampling effort as big as the data analyzed in the present study successfully detected population differentiation when *G*’_ST_ ≥ 0.01 (Power = 97.5% and 94.5% for *Χ*^2^ and Fisher’s exact tests, respectively). The bigger the sample size, the weaker the level of genetic divergence at which population differentiation was detected with power ≥ 95% (Figure 3). For instance, sampling 30 individuals, which is possible in well-preserved populations of *P. ferruginea*, could detect population differentiation when *G*’_ST_ ≥ 0.0025. Noteworthy, increasing the sampling effort to 50 individuals did not allow to detect with sufficient power the weakest level of genetic divergence considered in this set of simulations (*G*’_ST_ ≥ 0.001, Power < 80% for both tests).

**Figure 4:**
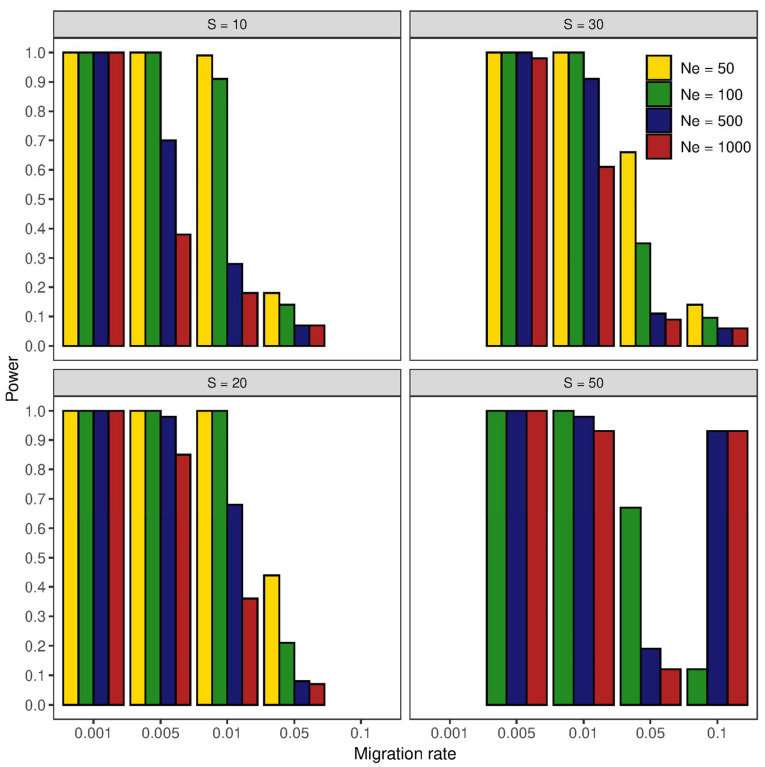
Sample size effect on the power of detecting population differentiation under different *N*e*m* values. Scenarios were simulated by combining different sample sizes (*S*), effective population sizes (*N*_e_), and migration rates (*m*) under a simple symmetric finite island model of gene flow. The power of detecting genetic differentiation was assessed by computing the proportion of successful detections over 1000 iterations for each scenario.

The results outlined above, based on the assumption that genetic drift was the only evolutionary force driving genetic divergence, were compared to those of a simple symmetric finite island model with varying effective population sizes (*N*_e_) and migration rates (*m*). The combination of these demographic parameters with different sampling efforts resulted in 60 simulated scenarios (Figure 4), which showed equilibrium *F*_ST_ values that ranged between 0.0001 and 0.32, encompassing those observed in the data (Table 3). Furthermore, as simulated *F*_ST_ values represented the overall mean among 10 populations, 44 out of 60 scenarios showed *F*_ST_ values that were included within the 95% confidence interval of the observed global *F*_ST_. Among these scenarios, only a quarter (11 out of 44) successfully detected population differentiation when genetic divergence was weak to moderate (*F*_ST_ = 0.004–0.023, Power ≥ 95%). Among these, only one scenario successfully detected population differentiation when the sample size was small (*S* = 10): this simulation combined a large effective population size (*N*_e_ = 1000) with a small migration rate (*m* = 0.001), resulting in an *F*_ST_ = 0.023. However, another scenario with *S* = 10 detected genetic differentiation showing a good success rate (Power = 91%): this simulation resulted in an overall *F*_ST_ = 0.022 by combining *N*_e_ = 100 and *m*= 0.01. Disregarding the magnitude of genetic differentiation, two further points need to be stressed. First, small sample sizes *per se* did not always jeopardize the success of detecting population differentiation: half of the scenarios simulated at *S* = 10 showed good to high power (≥90 and 95%, respectively). Power < 90% included scenarios characterized by large *N*_e_ and/or high *m* (Figure 4), which is an unlikely picture for most populations of *P. ferruginea*. Finally, increasing *S* increased the power of detecting population differentiation: for instance, a sampling effort of 50 individuals per population could successfully detect genetic differentiation when *F*_ST_ was as small as 0.0001. indeed, power = 93% in two scenarios with both large effective population size (*N*_e_ = 500 and 1000) and high migration rate (*m* = 0.1). However, the increase of power with sample size was not always linear as it can be shown in Figure 4: the power of detecting population differentiation did not substantially improve in some scenarios even when *S* was twice or five-fold larger than the smallest one.

## 4. Discussion

Restocking depauperate populations of *Patella ferruginea* by translocations or aquaculture is still a challenging practice and thus conservation should be yet focused on the safeguarding of the remaining well-preserved populations as well as the design of MPA networks [30,44,64]. Genetic tools may help fulfill these goals in two ways: (1) the monitoring of the conservation status and health of protected populations, and (2) the identification of those populations that maximize both genetic diversity and genetic uniqueness when designing MPA networks [65]. The latter goal might imply the screening of populations for which it could be difficult to obtain sufficiently large sample sizes. Here, empirical and simulated data are used to assess the reliability of genetic metrics useful to achieve the second goal when the sample size is small.

### 4.1. Study Limitations

Small sample sizes affect the precision and accuracy of allele frequency estimates and hence all the metrics relying on them are biased to an extent that depend on marker polymorphism [18]. However, expected and observed heterozygosity are less affected than allelic diversity by this bias, and unbiased expected heterozygosity shows good precision even when sample sizes are small [18,19]. Although allelic diversity underestimates genetic diversity with a bias that is proportional to the true effective population size, this metric is still useful to draw inferences from comparisons between populations with different levels of genetic diversity [17,19]. Also, our simulations support this trend, even though our results hold for close, isolated populations, which could be a realistic scenario only for some populations considered in the present study. Migration may slow down or counterbalance the rate at which alleles are lost, as in a metapopulation system the global effective population size is greater than the local population sizes [66].

Clues in on the effective population sizes that populations of *P. ferruginea* might currently have or might have had in the past are difficult to envisage. Yet, the simulated population sizes fall within the range observed in many marine organisms [14]. Moreover, effective population sizes ranging between 500 and 1000 individuals could be feasible in populations of *P. ferruginea* as large as those occurring along North African coasts [31]. In this case, the effective population size to abundance (*N*_C_) ratio will fall close to the upper bound (*N*_e_/*N*_C_ ratio = 10^−2^–10^−6^) usually observed in broadcast spawning marine species [67]. However, estimated contemporary *N*_e_ at Asinara and Tavolara MPAs indicate that *N*_e_/*N*_C_ ratio could be even higher than 10^−2^ in *P. ferruginea* [36], which is also consistent with recently revised estimates of this ratio in marine species [68]. Furthermore, large *N*_e_/*N*_C_ ratios may depend on gene flow among populations, for which local population sizes can be closer to the global metapopulation size [69], resulting in larger than expected *N*_e_.

Bottlenecks have been simulated assuming overlapping generations and a monoecious mating system without selfing as a proxy for the protandric sequential hermaphroditism of *P. ferruginea*. This choice ensures individuals behave as either males or females, thus mimicking the sex change mode of *P. ferruginea*: depending on environmental conditions, individuals may switch from males to females and back more times during their lifespan [30,38,40,42]. However, males and females will appear simultaneously in the simulated population, whereas nearly all individuals are males in real populations at sexual maturity but see [30]. Moreover, the sex ratio will likely be balanced during the entire forward simulations: conversely, real populations show male-biased sex ratios, which may range from 2:1 to 25:1 and vary over space, time, and size class [30,34,38]. As the unbalanced sex ratio further decreases *N*_e_, it cannot be ruled out that loss of genetic diversity could be larger in real than in simulated populations, all else being equal. The maximum duration of lifespan was set at 20 years, which is partly a subjective choice. Determining the age of limpets based on their shell size is not straightforward as it depends on the growth rate and the used method [27,30]. Hence, the lifespan was thus chosen to lie between 12 [30] and 35 years [44].

The framework used to model genetic differentiation relies upon simple population genetic models (pure genetic drift and symmetric finite island model) relative to seascape dispersal patterns and the demographic history of *P. ferruginea*. Yet, both models are suitable to assess the power of detecting population differentiation, even if they do not exhaustively account for asymmetric dispersal patterns, overlapping generations, and varying population sizes [16]. We considered models with and without migration because some populations considered in the present study are unlikely to be completely isolated, even though the most conservative estimates of genetic connectivity are assumed [36]. Remarkably, scenarios that combined small *N*_e_ with weak to moderate migration rates show a degree of genetic divergence that is twice up to more than a factor larger than observed in real populations. Such a discrepancy should not be interpreted as evidence that populations considered in the present study are characterized by larger than expected *N*_e_ and/or *m*. Population genetic models compute genetic divergence measures expected at migration-drift equilibrium, assuming that both *N*_e_ and *m* are constant over generations. This picture is unlikely in most natural populations and in those of *P. ferruginea* as well, which probably faced a gradual decline until the end of the 19th century, followed by the most severe size reductions during the last century [30]. Therefore, it cannot be ruled out that current levels of genetic divergence still reflect more historical levels of gene flow and population connectivity rather than a recent history of habitat fragmentation and population decline.

### 4.2. Effect of Sample Size on Genetic Diversity

Clearly, small sample sizes affect the magnitude of mean allelic richness (*A*_R_) as the probability of missing rare alleles increases. Hence, this metric is smaller than that reported in other limpets and gasteropods [5,70,71]. This point is further highlighted by the comparison between sites from Asinara (APS, ACS, APB) and Tavolara MPAs (MLA, MLT) with *A*_R_ estimates that were computed on the same MPAs but using large sample sizes [36]. In the latter case, *A*_R_ is as large as, or even larger than values observed in other coastal gasteropods. Nevertheless, the effect of sample size on *A*_R_ cannot be generalized to all sites, as exploitation might influence this metric as well. For instance, harvested limpet populations from the Macaronesian archipelago, *P. candei* and *P. aspera*, show levels of allelic richness that are comparable to those reported in the present study [72,73].

Conversely, small sample sizes do not affect the expected heterozygosity (*H*_E_), which is not surprising as the contribution of rare alleles to this metric is downweighed by their relative frequency. Hence, unsampled rare alleles will have a negligible impact on *H*_E_. Consistent with this picture, *H*_E_ values are similar to those reported for other gasteropods [5,70,71,73]. In contrast to other limpets, however, *P. ferruginea* does not show a heterozygote deficit as the magnitude of observed heterozygosity (*H*_O_) is as big as that of *H*_E_. this outcome might partly be a side-effect of small sampling effort, as *H*_O_ is slightly smaller than *H*_E_ when large samples can be analyzed [36]. Likely, population decline might partly underpin the slight to moderate heterozygote excess observed at most sites (Table 2). Indeed, tiny population sizes may determine a heterozygote excess (*H*_E_ < *H*_O_, *F*_IS_ < 0) by increasing the rate at which rare alleles are lost [74].

The patterns outlined above are well depicted by simulations that represent the effect of population size reductions on genetic diversity (Figure S1). As expected, the number of alleles shows a larger decrease than other metrics, regardless of the presence of a bottleneck and/or its severity. Indeed, metrics based on allelic diversity are more sensitive than expected and observed heterozygosity to population size changes, as evidenced by both empirical and simulated data, and may reflect more effectively the species’ evolutionary potential, as well [12,17]. Random sub-sampling on simulated data with different population sizes, which will be hereafter considered as proxies for protected/inaccessible (large size, *N*_e_ = 500) or unprotected/exploited populations (moderate and small size, *N*_e_ = 100 and 50, respectively), further highlights some interesting remarks. A small sample size does not preclude the comparison of allelic diversity between genetically diverse (populations with no history of bottlenecks because of enforced protection or inaccessibility) and depauperated populations (which suffer bottlenecks because of exploitation). Although allelic diversity is underestimated throughout all scenarios, large-sized populations always show a mean allelic richness that is higher than that of small-sized ones at all levels of sampling. For instance, Figure 2 illustrates a case in which the mean allelic richness of the unbottlenecked population is nearly twice that of a ten-fold bottlenecked one even when the sample size is very small (S ≥ 8). Moreover, the variance associated with a small sample size will not affect the power of detecting differences in allelic diversity as 95% confidence intervals, which outline the uncertainty of allelic diversity estimates, do not overlap. Results based on simulations (see also Figure S2) match well those based on both empirical data of genetically diverse and depauperated populations of a songbird and computer simulations [17,19]: whatever the sample size, the most genetically diverse populations always showed the highest levels of genetic diversity.

Simulated data of *P. ferruginea* showcased in Figure 2 and Figure S2 also illustrate another point outlined by empirical data [18,19,36]: at least 30 individuals need to be sampled to approach the maximum value of allelic diversity. However, when the true effective size is moderate (*N*_e_ = 100) or large (*N*_e_ = 500), even more individuals are needed (*S* ≥ 40) to include the maximum value of allelic diversity within the confidence interval of random sub-samples. Nonetheless, even sample sizes commonly used in microsatellite datasets (40–50 individuals per population) could not be sufficient to include the true value of allelic diversity in the confidence interval when *N*_e_ is large. Empirical data on populations of *P. ferruginea*, whose *N*_e_ likely ranged between 100 and 500 individuals, further highlight this point [36], thus confirming that no reasonable sample sizes will likely ensure the detection of all the rare alleles in populations with sufficiently large effective sizes [17].

The severity of population size reduction slightly affects the power of detecting differences in allelic diversity between populations with or without a history of bottlenecks. Sampling effort should be doubled (*S* ≥ 16) to safely detect differences in allelic diversity between a large sized-population and one that is undergoing a five-fold population size reduction, relative to the former (Figure 2). The same relationship, however, does not hold when comparing moderately and severely bottlenecked populations (Figure S2): hence, caution is needed in drawing inferences based on the comparison of allelic diversity estimates if weak or moderate population size declines are suspected.

Alternatively, allelic diversity can be extrapolated from small sample sizes enforcing, for instance, non-linear regression models [75]. Besides the fact that extrapolation is less precise and accurate than random subsampling and rarefaction [17], this method needs densely sampled populations to accurately predict values of genetic diversity. Moreover, the accumulation of allelic diversity at increasing sample sizes must implicitly follow the same model for all populations. This condition is likely met when populations have roughly similar, large effective population sizes (Figure 2 and Figure S2). Therefore, non-linear models (or other methods) could be a feasible tool for extrapolating allelic diversity from well-preserved or protected populations with this caveat in mind: even small differences in *N*_e_, and likely the demographic history of populations, may lead to non-negligible differences of their accumulation curves [36]. Conversely, extrapolation methods should be cautiously adopted or even disregarded when obtaining large sample sizes is unfeasible, such as in non-protected, exploited populations, for which *N*_e_ is either unknown or small. Indeed, the accumulation of allelic diversity flattens more quickly in small- than large-sized populations and, depending on the effective population size, tends to vary more unpredictably (Figure S2).

### 4.3. Effect of Sample Size on Genetic Differentiation

In contrast to former studies [28,47], the present study does not evidence genetic structuring along Sardinian coasts (Table 3). However, this discrepancy does not entirely depend on the lack of power of microsatellites as the same markers can detect genetic divergence even on spatial scales as small as few km [36]. Extending the comparison to studies on other limpet species is not straightforward as both different sets of microsatellites, as well as different metrics of genetic divergence, were used. Nevertheless, the range of genetic divergence estimated in *P. ferruginea* is roughly similar to those reported in *P. caerulea*, *P. candei,* and *P. aspera* [70,72,73]. Interestingly, those studies found significant population differentiation when genetic divergence among population pairs exceeded 0.01, which is the threshold suggested to be used for the delimitation of demographically independent populations [14,55]. In the present study, Fisher’s exact test does not infer population differentiation notwithstanding that 21 and 34 out of 91 pairwise comparisons based on *ϴ* and *G*’_ST_, respectively, exceed the above threshold. Perhaps, this pattern reflects the fact that *F*_ST_ and analogous metrics are sensitive to undersampling, as simulation studies based on empirical data evidenced [76]. Small samples are less likely to detect subtle patterns of genetic differentiation because the reduced precision and accuracy of estimates decrease the statistical power of detecting differences [15,16]. The large 95% confidence intervals observed herein fit this picture (Table S4). Indeed, this result is consistent with the higher coefficient of variation of genetic differentiation observed in small compared to large samples [16].

Nevertheless, this issue mainly concerns large than small populations (i.e., large versus small *N*_e_): as *F*_ST_ will tend to be small in large populations, sufficiently large sample sizes are needed to increase the precision and accuracy of genetic differentiation estimates. Conversely, if *F*_ST_ is large as it could be in small populations, small sample sizes (fewer than 20 individuals if *F*_ST_ > 0.05) could be sufficient to obtain precise and accurate estimates of genetic differentiation [16]. Our simulations match the above trends, indicating that a small sample size is not always a hindrance to detecting population differentiation with highly polymorphic markers as microsatellites are. Furthermore, depending on the demographic history of populations (*N*_e_ and levels of gene flow), small samples can successfully detect population differentiation even when *F*_ST_ ≥ 0.01 (Figure 3 and Figure 4). Large samples are needed to assess population differentiation when genetic divergence is weak; yet, increasing the sample size has decreasing advantages, which may become negligible between 30 and 50 individuals. Once again, this trend matches well those reported in former studies [16,18]; however, the number of individuals required to assess population differentiation when *F*_ST_ < 0.01 is smaller than that envisioned by a previous study [16].

Although low statistical power because of the small sample size is the more likely explanation for the absence of genetic structure, two alternative scenarios cannot be ruled out, based on simulated data (and the known demographic history of *P. ferruginea*). First, as *F*_ST_ depends on both historical and contemporary gene flow [6], genetic patterns might be a snapshot of a recently shared evolutionary history. Since the most severe decline of the species began at the end of the 19th century, it cannot be disregarded that both the number and size of populations before this date were sufficient to ensure genetic connectivity, thus preventing genetic differentiation. Consistent with this picture, Microsatellite markers may need as many as 200 generations to reach 50% *F*_ST_ equilibrium values after the onset of isolation [77]. Nevertheless, the above evolutionary history is also hard to reconcile with the spatial genetic structuring retrieved by ISSR fingerprinting on the same individuals and populations [28]. Alternatively, genetic patterns might be driven by ongoing gene flow which is higher than expected. The larval cycle of *P. ferruginea*, which has been recently investigated by breeding individuals in aquaculture tanks, disclosed life-history traits that may underpin a dispersal potential of at least tens of km [30]. This being true, the recent institution of MPAs along the Sardinian coasts might already have had a positive effect on population connectivity, favoring source-sink dynamics. We may speculate that the resulting multi-generational genetic connectivity shall counterbalance the genetic drift due to small local genetic effective size by a larger global effective metapopulation size [66]. The genetic patterns resulting from such a process do not contrast with those retrieved by ISSRs. Indeed, if highly polymorphic markers require a large lag time to detect genetic differentiation using metrics such as *F*_ST_, the reverse does not hold true: when genetic connectivity is restored, footprints of genetic structuring are quickly erased in the lapse of a few generations [77]. Conversely, less variable markers such as ISSRs might have retained genetic signatures of past events for longer times.

### 4.4. Implications for the Conservation of Endangered Marine Species

Even when extensive sampling is possible, the best trade-off between sampling effort and power should be pursued for endangered and or rare species: sampling should be tailored to the minimum largest possible size [15]. The results of the present study, albeit the simulations have been tailored to the life-history traits of *P. ferruginea*, can be extended at least to marine species with similar biology. Overall, the outcomes match the trends reported in previous studies, which is not obvious for broadcast spawners, which rely upon larval stages to ensure connectivity. Our simulated data agree with the figures suggested previously [18]: 20–30 individuals are likely sufficient to accurately estimate allele frequency-based metrics such as allelic diversity, *F*_ST,_ or related measures of genetic differentiation.

Conversely, caution is needed when small sample sizes (fewer than 20 individuals) are unavoidable. Our results line up with former studies and indicate that metrics estimating genetic diversity within populations are still informative even when sample sizes are fairly small. For instance, unbiased expected heterozygosity seems to estimate genetic diversity with good precision even when the sample size is smaller than 10 individuals, as observed in songbird populations [19]. Allelic diversity could be used to compare differences in genetic diversity among populations, even if it underestimates the number of alleles when sample sizes are small. Consistently with former studies [17], [19], it will be particularly useful for comparing populations with no history of bottlenecks and those that underwent bottlenecks. In the specific case of *P. ferruginea*, for instance, it could be useful for comparing levels of genetic diversity between protected and non-protected populations, or between easily accessible and hardly accessible sites.

In contrast, assessing genetic differentiation could be a really difficult issue related to assessing population connectivity or the genetic uniqueness of populations. Results based on simulations show it is possible to infer population differentiation using small sample sizes, as well as it is unlikely to detect genetic structuring when it is absent. Therefore, when moderate to strong genetic differentiation is assessed, it should not be dismissed as unreliable just because of the small sample size, as recommended by Björklund and Bergek [15]. However, it is a more hard-to-solve question disentangling whether the absence of genetic structure depends on the evolutionary and demographic history of populations in that region or merely reflects low statistical power. If it is not possible to collect more samples, increasing the number of markers might alleviate this issue [16].

Our results also stress the importance of carrying genetic monitoring programs on well-preserved populations of endangered species. Our simulations show that large effective population size (*N*_e_ = 500 individuals) does not always ensure the maintenance of allelic diversity over ecological timescales as nearly 20% of alleles are lost over 100 years. This trend further supports the dismissal of the 50/500 rule in favor of the revised 100/1000 rule [78], which suggests to increase the thresholds of effective population size needed to avoid inbreeding in the short-term (*N*_e_ = 50 to 100) and maintain the evolutionary potential in the long-term (*N*_e_ = 500 to 1000). The use of the revised rule in future studies on *P. ferruginea*, however, should not disregard the following caveat: simulations concerned completely isolated populations. The global effective size of a network of populations connected by gene flow may be large enough to ensure both the short- and the long-term persistence of local populations, despite the local effective population sizes being small [15].

As a last remark, the multiplex tool here developed is best suited for the genetic monitoring of those populations of *P. ferruginea* in which large samples can be easily obtained without harming the whole population. Indeed, when the goal is focused on the conservation and health status of populations, estimating levels of recruitment, contemporary effective population size, or assessing recent bottlenecks, one should strive for sample sizes larger than 30 individuals, if it is possible.

## 5. Conclusions

The present study combines both empirical and simulated data to investigate how small sample sizes influence the detection of genetic patterns in marine endangered species. Although the study is focused on *Patella ferruginea*, results can be expanded to other marine species with similar life-histories. Overall, the present study outlines that valuable genetic information can be obtained even when sample sizes are small, depending on the species’ biology and demographic history. This outcome is important for decision-making in conservation plans dealing with endangered, rare, or elusive species, for which it is neither advisable nor feasible to achieve sufficiently large sample sizes. The following guidelines may be useful for conservation practitioners interested in embedding genetic information into conservation plans. First, high population connectivity should be inferred with caution when sample sizes are small and the species’ life-history traits are poorly known. Second, both expected heterozygosity and allelic richness should be used in conservation frameworks as these metrics give complementary information on patterns of genetic diversity. Finally, even seemingly well-preserved but potentially isolated populations may need genetic monitoring in order to prevent the loss of species’ adaptive and evolutionary potential over time.

## Figures and Tables

**Table 1:** Characteristics of the microsatellite loci used in the present study. F = Forward sequence; R= reverse sequence; bp = base pairs. See Table S2 for protocol details.

Primer	Sequence	Repeat Motif	Multiplex (Fluorescent Dye)	Size Range (bp)	Genbank Accession Number
Pf-31AH8	F: GGGTGTGGCTCTGCCTATTA R: TGGTTACCCCAGATATACGC	(CA)2 GA (CA)8	MpxB (6-FAM)	123–131	FJ436433
Pf-31IF2	F: TATCCTACATACCATCCATAATGC R: TAGTCCATAGTGCCGCTGTC	(CA)12	(NED) ^1^	188–216	FJ436435
Pf-D11A	F: ACAACGAAGCCACCGACTAC R: AGCGCACTTCTTGACCTGAC	(CT)13	MpxA (VIC)	386–426	FJ436436
Pf-G1M	F: GGCTCAGTTCGAGAATCCAC R: TAACCGACCATTCACGTGTT	(CA)15	MpxA (6-FAM)	143–177	FJ436437
Pf-C10	F: TCTATGCTAATATTTGTGTCTGTCG R: TTCACCCGGCTAAAGAATCA	(TYTG)3 TCG (TYTG)4 TTG (TYTG)4	MpxB (6-FAM)	312–328	FJ436438
Pf-G6A	F: CCAAATAGTCTTCGTGGTTGG	(TG)15 GG (TG)8	MpxB (VIC)	225–251	FJ436439
Pf-31IB2	F: TGGATAGTGGGTATGTGTTGC R: GTAGCCACCAATCCATTAGC	(GT)14	MpxA (NED)	90–114	FJ436441
Pf-31IB1	F: GTTGTTGCGATTTCATGTGG R: TTAAGAATTGTGGCCTGTTGG	(TG)21 (TATG)3 (TG)2 (TC) (TG)2	MpxA (VIC)	124–192	FJ436443

^1^ Due to poor multiplex amplification this locus was removed from MpxB.

**Table 2:** Summary statistics of within-population genetic variation averaged over loci. Populations are abbreviated as in Figure 1. *A*: number of alleles; *A*_R_: allelic richness; *A*_E_: number of effective alleles; *H*_E_: expected heterozygosity; *H*_O_: observed heterozygosity; u*H*_E_: unbiased expected heterozygosity; *F*_IS_ = inbreeding coefficient; SE: Standard Error.

Population	*A* ± SE	*A*_R_ ± SE	*A*_E_ ± SE	*H*_E_ ± SE	*H*_O_ ± SE	u*H*_E_ ± SE	*F*_IS_ ± SE
APS	5.12 ± 1.11	3.86 ± 0.69	3.37 ± 0.73	0.55 ± 0.11	0.53 ± 0.11	0.58 ± 0.12	0.02 ± 0.05
ACS	4.37 ± 0.71	3.72 ± 0.52	3.15 ± 0.50	0.58 ± 0.10	0.60 ± 0.11	0.62 ± 0.11	−0.05 ± 0.06
APB	5.62 ± 1.05	4.51 ± 0.77	4.13 ± 0.86	0.63 ± 0.10	0.64 ± 0.11	0.68 ± 0.11	−0.03 ± 0.07
CDN	6.25 ± 1.25	4.64 ± 0.65	4.21 ± 1.00	0.62 ± 0.10	0.67 ± 0.11	0.65 ± 0.11	−0.07 ± 0.04
IPO	5.00 ± 0.91	3.98 ± 0.56	3.44 ± 0.61	0.61 ± 0.10	0.67 ± 0.12	0.64 ± 0.10	−0.11 ± 0.07
ARG	6.12 ± 1.06	4.35 ± 0.43	3.62 ± 0.62	0.62 ± 0.10	0.63 ± 0.11	0.64 ± 0.10	−0.02 ± 0.04
MVE	5.75 ± 1.11	4.52 ± 0.71	4.27 ± 0.85	0.65 ± 0.10	0.73 ± 0.11	0.70 ± 0.11	−0.14 ± 0.04
CPA	5.37 ± 1.02	4.09 ± 0.57	3.66 ± 0.68	0.60 ± 0.11	0.64 ± 0.12	0.64 ± 0.11	−0.08 ± 0.05
CPC	5.75 ± 1.08	4.34 ± 0.61	3.93 ± 0.78	0.64 ± 0.09	0.66 ± 0.11	0.67 ± 0.09	0.01 ± 0.10
PLF	5.25 ± 0.96	4.22 ± 0.69	3.94 ± 0.82	0.61 ± 0.11	0.69 ± 0.13	0.65 ± 0.12	−0.13 ± 0.06
MAD	5.00 ± 0.84	4.09 ± 0.64	3.64 ±0.71	0.59 ± 0.11	0.66 ± 0.12	0.63 ± 0.11	−0.12 ± 0.06
CAM	5.50 ± 0.96	4.16 ± 0.56	3.63 ± 0.61	0.61 ± 0.11	0.62 ± 0.11	0.65 ± 0.11	−0.03 ± 0.06
MLA	5.25 ± 1.01	4.31 ± 0.60	3.78 ± 0.65	0.63 ± 0.10	0.63 ± 0.11	0.67 ± 0.11	0.00 ± 0.05
MLT	5.37 ± 1.02	4.31 ± 0.64	3.54 ± 0.73	0.59 ± 0.10	0.62 ± 0.11	0.63 ± 0.11	−0.05 ± 0.05

**Table 3:** Pairwise population differentiation. Weir and Cockerham’s *ϴ* and Hedrick’s *G*’_ST_ values are reported below and above the diagonal, respectively. Values outlined in bold indicate population pairs showing significant genetic divergence based on Fisher’s exact test for genetic homogeneity of genotype frequencies. Due to multiple testing, probability values were adjusted following Narum [56]. Populations are abbreviated as in Figure 1.

	APS	ACS	APB	CDN	IPO	ARG	MVE	CPA	CPC	PLF	MAD	CAM	MLA	MLT
APS	—	0.041	0.018	−0.004	0.014	0.037	0.013	0.035	−0.008	0.010	0.042	0.006	0.017	0.066
ACS	0.016	—	−0.060	0.019	0.009	0.026	−0.009	0.047	0.028	−0.024	−0.044	−0.016	0.008	0.103
APB	0.003	−0.029	—	−0.045	−0.008	0.015	−0.069	−0.042	−0.042	−0.041	−0.039	−0.086	−0.059	0.038
CDN	−0.003	0.008	−0.018	—	−0.064	−0.016	−0.014	0.011	−0.030	−0.052	−0.002	−0.040	−0.048	0.036
IPO	0.005	0.005	−0.003	−0.026	—	−0.006	−0.016	0.016	0.006	−0.027	0.021	−0.036	0.000	0.027
ARG	0.015	0.010	0.005	−0.007	−0.002	—	0.012	0.077	−0.009	0.005	0.022	0.007	−0.020	0.041
MVE	0.005	−0.002	−0.026	−0.003	−0.002	0.006	—	−0.001	−0.010	0.001	−0.006	−0.057	−0.019	0.052
CPA	0.014	0.021	−0.018	0.006	0.009	0.033	0.002	—	0.012	−0.010	0.000	−0.032	0.023	0.069
CPC	−0.007	0.011	−0.019	−0.012	0.003	−0.004	−0.002	0.005	—	−0.032	0.005	−0.019	−0.008	0.009
PLF	0.004	−0.010	−0.016	−0.020	−0.008	0.003	0.005	−0.002	−0.012	—	−0.050	−0.019	−0.018	0.050
MAD	0.018	−0.020	−0.017	0.001	0.013	0.010	0.002	0.001	0.003	−0.019	—	−0.039	−0.013	0.069
CAM	−0.002	−0.011	−0.039	−0.018	−0.016	0.002	−0.023	−0.015	−0.009	−0.008	−0.018	—	−0.058	0.041
MLA	0.004	0.001	−0.026	−0.020	0.000	−0.009	−0.007	0.009	−0.005	−0.007	−0.006	−0.027	—	0.017
MLT	0.029	0.045	0.014	0.016	0.013	0.018	0.023	0.030	0.003	0.023	0.031	0.016	0.006	—

## Data Availability

The data presented in this study and the R-scripts used for data analysis are openly available in FigShare at https://doi.org/10.6084/m9.figshare.14611407.v1. R-scripts used for graphics are available by the corresponding author upon reasonable request.

## Supplementary Materials

The following supporting information can be downloaded at: https://www.mdpi.com/article/10.3390/ani12202763/s1, Figure S1: Results of bottleneck simulations; Figure S2: Effect of sample size on genetic diversity; Table S1: Sampling locations of *Patella ferruginea*; Table S2: Protocol of multiplex PCR reactions; Table S3: Allele frequencies for each locus within sites retained for the analysis; Table S4: Pairwise genetic differentiation.
