# Supplementary material for: Detection of Genetic Patterns in Endangered Marine Species Is Affected by Small Sample Sizes
Source: Animals (Basel). 2022 Oct 14;12(20):2763. doi: 10.3390/ani12202763 (PMC9597844; doi:10.3390/ani12202763)
Supplement: Supplementary file 1 [file animals-12-02763-s001.zip › Supplementary figures.pdf]

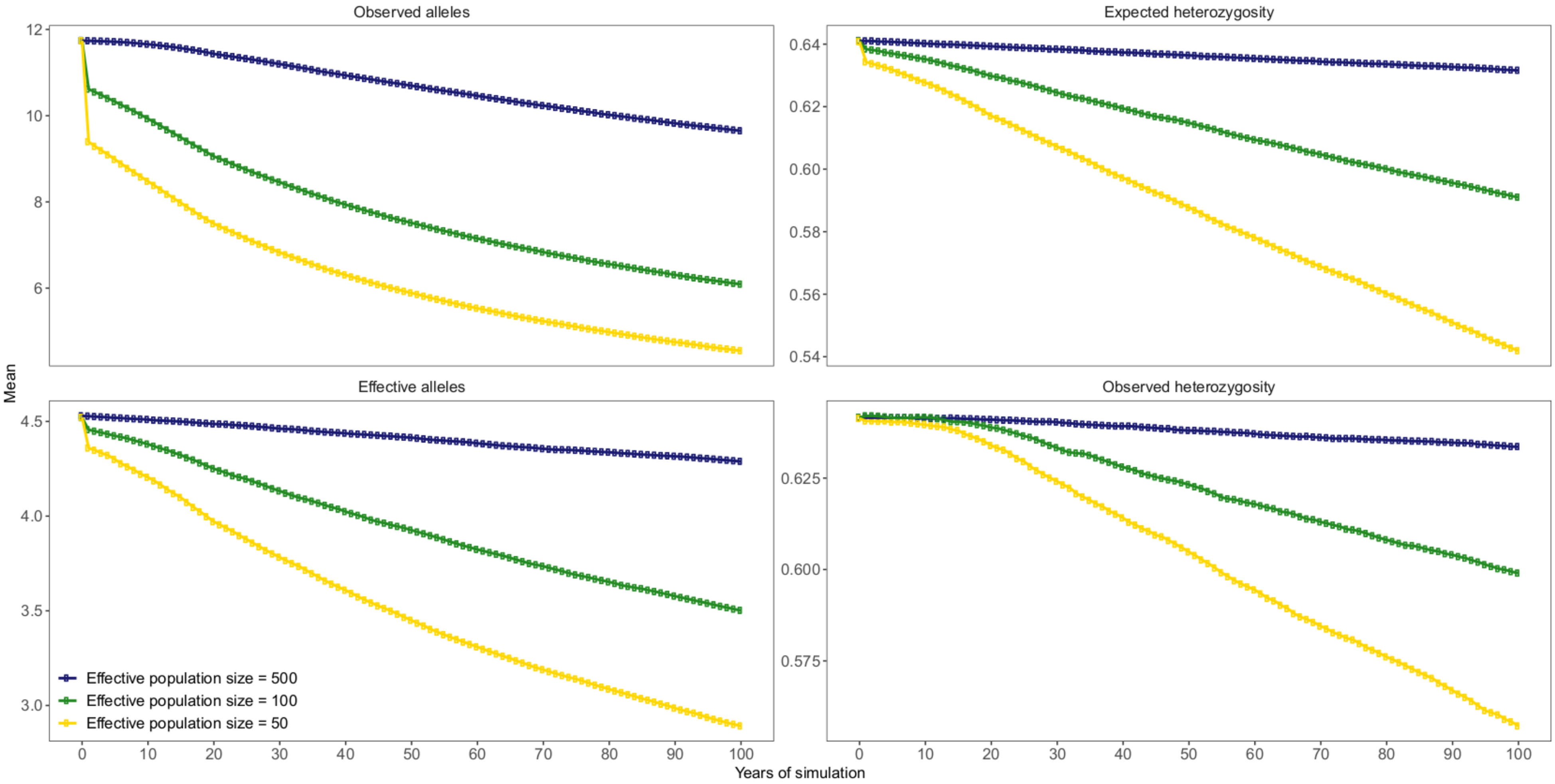

Figure S1. Results of Bottleneck simulations. For each scenario, the evolution of four metrics of within population genetic diversity is depicted over 100 years. At year zero all populations have the same population effective size (N_e = 500 individuals), after which (year 1) populations undergo: no size reduction (N_e = 500); moderate (five-fold) size reduction (*Ne* = 100); severe (10-fold) size reduction (*Ne* = 50). Such effective population sizes remain constant for the remainder of the simulations.

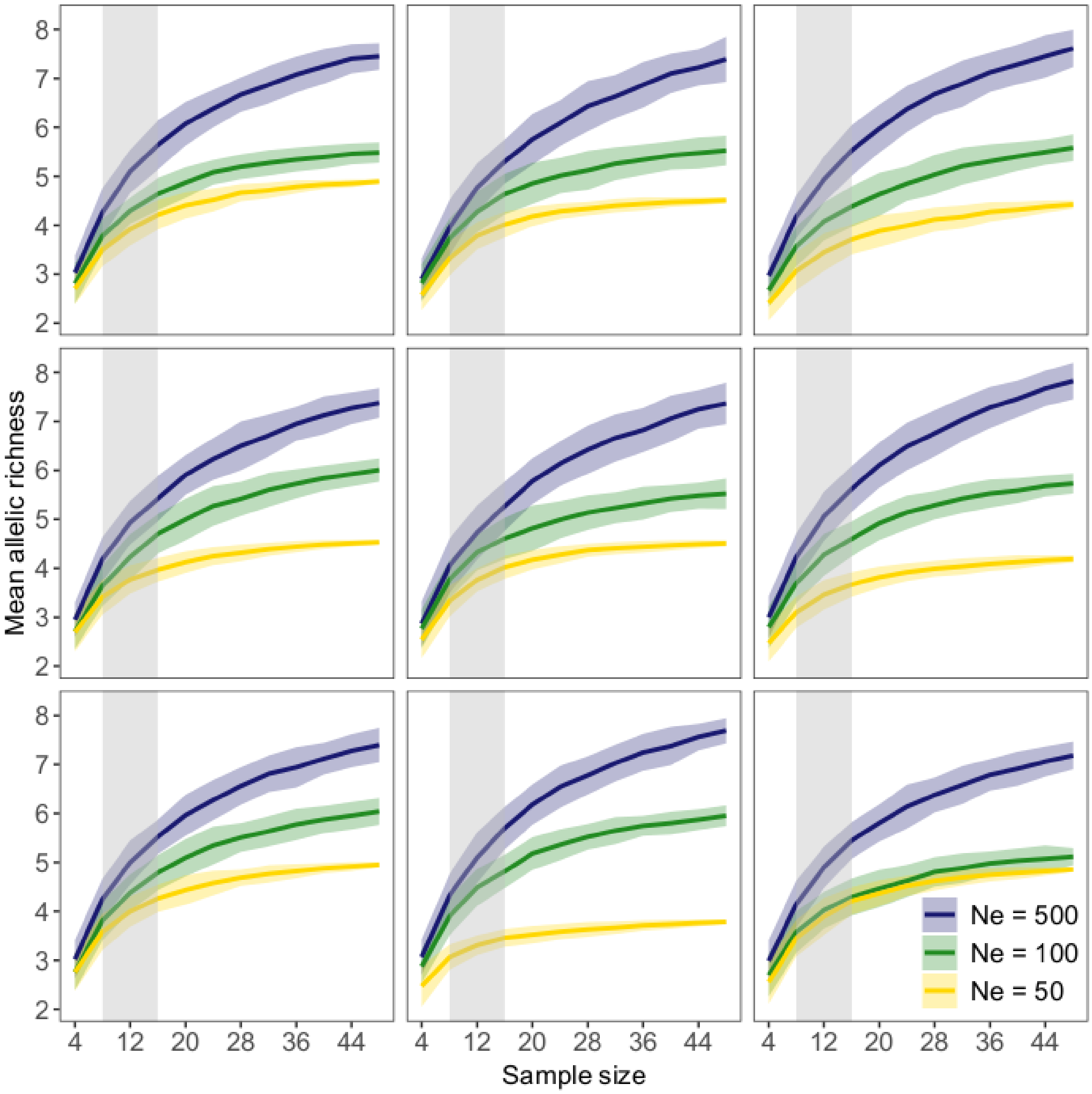

Figure S2. Effect of sample size on genetic diversity. Solid lines represent the mean value of allelic richness at increasing sample size for three populations with different effective population size. Shaded areas around each line outline the 95% confidence interval estimated on 100 resampled datasets. The gray shaded area encompasses the sample size interval of the real data.
