# Supplementary material for: Detection of Genetic Patterns in Endangered Marine Species Is Affected by Small Sample Sizes
Source: Animals (Basel). 2022 Oct 14;12(20):2763. doi: 10.3390/ani12202763 (PMC9597844; doi:10.3390/ani12202763)
Supplement: Supplementary file 1 [file animals-12-02763-s001.zip › Supplementary tables.pdf]

Table S1. Sampling locations of *Patella ferruginea*. Note that the Western Mediterranean marine ecoregion (ME) does not match with the Western basin of the Mediterranean Sea, which encompass the Alboran sea, Western Mediterranean and part of the Tunisian plateau-Gulf of Sydra MEs. *S* = sample size

Label	Site	Locality	Marine ecoregion	*S*	Longitude	Latitude
APS	Punta Sabina	Asinara island	Western Mediterranean	9	8.349694	41.08929
ACS	Cala Sant'Andrea	Asinara island	Western Mediterranean	8	8.24928	41.01457
APB	Pedra Bianca	Asinara island	Western Mediterranean	7	8.211542	41.00374
CFA	Capo Falcone	Stintino	Western Mediterranean	1	8.202004	40.97743
CDN	Coscia di Donna	Stintino	Western Mediterranean	11	8.219403	40.90023
IPO	Isola dei Porri	Stintino	Western Mediterranean	9	8.214456	40.88156
ARG	Argentiera	Sassari	Western Mediterranean	16	8.147306	40.72519
AHO	Alghero	Alghero	Western Mediterranean	4	8.146247	40.6077
MVE	Mal di Ventre Island	Oristano	Western Mediterranean	8	8.302379	39.99454
PTO	Porto Torres	Porto Torres	Western Mediterranean	1	8.417213	40.83866
CPB	Costa Paradiso B	Isola Rossa	Western Mediterranean	5	8.932825	41.0479
CPA	Costa Paradiso A	Costa Paradiso	Western Mediterranean	10	8.946617	41.05681
CPC	Costa Paradiso C	Costa Paradiso	Western Mediterranean	10	8.968165	41.07772
PLF	Punta Li Francesi	Vignola	Western Mediterranean	8	9.051474	41.13606
MAD	Madonnetta	La Maddalena	Western Mediterranean	8	9.376367	41.23067
CAM	Isola delle Camere	Porto Rotondo	Western Mediterranean	9	9.584939	41.06928
MLA	Molara Island	Olbia	Western Mediterranean	9	9.72962	40.87656
MLT	Molarotto island	Olbia	Western Mediterranean	9	9.778789	40.87431
GAL	Galeria	Galeria	Western Mediterranean	3	8.652303	42.41547
CAR	Cargese	Cargese	Western Mediterranean	1	8.587172	42.12896
TIZ	Tizzano	Tizzano	Western Mediterranean	1	8.850947	41.53868
DIQ	Dique de Poniente	Ceuta	Alboran Sea	3	-5.31834	35.89823
DES	Desnarigado	Ceuta	Alboran Sea	2	-5.28121	35.89405
CRL	El Chorrillo	Ceuta	Alboran Sea	2	-5.32378	35.88134
MEL	Melilla	Melilla	Alboran Sea	3	-2.95588	35.32451
CHA	Chafarinas island	Ras Kebdana	Alboran Sea	4	-2.43172	35.18168
HAB	Habibas island	Oran	Alboran Sea	5	-1.13521	35.71968
PLA	Plane island	Oran	Alboran Sea	2	-0.90185	35.7717
CAP	Cape Bon	Cape Bon	Tunisian Plateau-Gulf of Sydra	5	11.03829	37.08637

Table S2. Deatails on the PCR (Polymerase Chain Reaction) protocol of the two multiplex reactions. For each component of the PCR mixture,the initial concentration is outlined between parentheses. Microsatellite amplification ofeach DNA sample was carried out in a final volume of 25 µl.

**Amplification protocol**			
**Polimerase Chain Reaction (PCR) mixture**			
**Reagents**	**Volume for one sample reaction (µl)**		
**Multiplex A (MpxA)**			
DNA[(a)]	2		
Buffer (10X)	2.5		
$MgCl_2$ (50 mM)	1		
dNTPs (10 mM)	0.5		
Pf-D11A Forward primer (10 µM)	0.7		
Pf-D11A Reverse primer (10 µM)	0.7		
Pf-G1M Forward primer (10 µM)	0.5		
Pf-G1M Reverse primer (10 µM)	0.5		
Pf-31IB2 Forward primer (10 µM)	0.4		
Pf-31IB2 Reverse primer (10 µM)	0.4		
Pf-31IB1 Forward primer (10 µM)	0.5		
Pf-31IB1 Reverse primer (10 µM)	0.5		
Taq polymerase (5u/µl)	0.2		
H_2O	14.6		
**Multiplex B (MpxB)**			
DNA[(a)]	2		
Buffer (10X)	2.5		
$MgCl_2$ (50 mM)	1		
dNTPs (10 mM)	0.5		
Pf-31AH8 Forward primer (10 µM)	0.4		
Pf-31AH8 Reverse primer (10 µM)	0.4		
Pf-C10 Forward primer (10 µM)	0.5		
Pf-C10 Reverse primer (10 µM)	0.5		
Pf-G6A Forward primer (10 µM)	0.4		
Pf-G6A Reverse primer (10 µM)	0.4		
Taq polymerase (5u/µl)	0.2		
H_2O	16.2		
**Thermal cycler amplification profile**			
**Step**	**Temperature**	**Time**	**Cycles**
Initial denaturation	94°C	5 minutes	1
Denaturation	94°C	50 seconds	
Annealing	54°C	50 seconds	35
Extension	72°C	50 seconds	
Finalextension	72°C	30minutes	1

(a) Each reaction contained 20-30 ng of genomic DNA

Table S3. Allele frequencies for each locus within sites retained for the analysis. Each allele is labelled according to the fragment size in base pairs. Site labels are as in Table S1

Locus	Allele	APS	ACS	APB	CDN	IPO	ARG	MVE	CPA	CPC	PLF	MAD	CAM	MLA	MLT
Pf-31AH8	123			0.071	0.045		0.031			0.150					
	129	1.000	1.000	0.929	0.955	1.000	0.969	1.000	1.000	0.850	1.000	1.000	1.000	1.000	1.000
Pf-31IF2	188	0.056	0.250	0.143	0.182	0.333	0.094		0.250	0.050	0.214	0.125	0.111		
	190	0.056	0.063		0.091	0.111	0.250	0.125	0.050	0.050	0.071	0.063	0.167	0.214	0.188
	192	0.222	0.375	0.286	0.182	0.111	0.250	0.375	0.200	0.300	0.214	0.250	0.278	0.286	0.125
	194	0.556	0.188	0.286	0.500	0.389	0.375	0.250	0.450	0.500	0.357	0.438	0.389	0.500	0.500
	196							0.063	0.050		0.143	0.063	0.056		
	210	0.056	0.125	0.214	0.045	0.056	0.031	0.188		0.050		0.063			0.188
	212	0.056								0.050					
	214			0.071											
Pf-D11A	388			0.071											0.056
	390				0.045										0.056
	394				0.045	0.063				0.050				0.063	
	396							0.071							
	398	0.167	0.250	0.071	0.091	0.188	0.100	0.071	0.050		0.188	0.063			0.056
	400	0.056			0.091	0.125	0.033	0.071	0.100		0.063		0.125		
	402	0.167		0.071	0.136	0.125	0.033	0.071		0.050	0.125	0.188	0.125	0.063	
	404	0.056		0.071	0.136	0.063	0.067	0.071		0.150	0.063		0.063	0.063	0.056
	406	0.056	0.083	0.071	0.045	0.063	0.033	0.071	0.100	0.050	0.063		0.063		0.111
	408	0.111	0.083	0.071	0.136	0.063	0.167	0.214	0.050	0.100	0.125	0.188	0.125	0.125	0.056
	410	0.167	0.167	0.357	0.136	0.250	0.167	0.143	0.350	0.150	0.125	0.188	0.313	0.375	0.222
	412	0.222	0.250	0.214	0.045		0.267	0.071	0.200	0.250	0.125	0.188	0.063	0.125	0.167
	414				0.045			0.071	0.150	0.100		0.063	0.063		0.111
	416		0.167		0.045	0.063	0.067				0.125	0.125	0.063	0.188	
	418						0.067	0.071		0.050					
	420														0.111
	424									0.050					
Pf-G1M	143	0.444	0.125	0.143	0.318	0.333	0.375	0.188	0.167	0.350	0.313	0.125	0.222	0.278	0.167
	149	0.444	0.375	0.286	0.273	0.389	0.250	0.375	0.389	0.350	0.313	0.375	0.278	0.222	0.500
	151	0.111	0.250	0.429	0.182	0.056	0.125	0.188	0.222	0.200	0.188	0.125	0.333	0.222	0.111
	153			0.071	0.136	0.167	0.156	0.125	0.056		0.063	0.063	0.056	0.111	0.056
	155							0.063	0.056	0.050					
	159		0.188	0.071	0.091	0.056	0.094		0.111	0.050	0.125	0.250	0.111	0.167	0.167
	161		0.063									0.063			
	173							0.063							
Pf-C10	312	0.056	0.188	0.143	0.091	0.125	0.094	0.250	0.150	0.150	0.063	0.188	0.111	0.167	0.111

Locus	Allele	APS	ACS	APB	CDN	IPO	ARG	MVE	CPA	CPC	PLF	MAD	CAM	MLA	MLT
	316				0.045	0.125	0.094	0.063			0.063		0.056	0.111	0.056
	328	0.944	0.813	0.857	0.864	0.750	0.813	0.688	0.850	0.850	0.875	0.813	0.833	0.722	0.833
Pf-G6A	225								0.050						
	227						0.094	0.063						0.056	0.056
	229	0.167	0.125	0.143	0.091	0.056		0.188	0.050		0.063	0.125	0.111	0.222	0.111
	231	0.333	0.625	0.429	0.409	0.444	0.438	0.375	0.350	0.400	0.500	0.688	0.500	0.333	0.167
	233	0.111	0.125	0.143	0.136	0.278	0.125	0.250	0.300	0.200	0.188	0.125	0.167		0.111
	235	0.056	0.125	0.143	0.091		0.031		0.050	0.050		0.063	0.111	0.111	0.056
	237	0.222		0.071	0.227	0.222	0.281	0.063	0.100	0.200	0.188		0.056	0.222	0.444
	239	0.056		0.071						0.150					0.056
	241												0.056	0.056	
	243	0.056					0.031	0.063	0.100						
	245				0.045										
	247										0.063				
Pf-31IB2	90	0.111	0.063	0.071	0.045		0.031	0.125				0.063		0.056	
	92									0.050					
	94		0.125	0.143	0.136	0.222	0.094	0.125	0.200	0.200	0.188	0.188	0.167	0.111	0.444
	96			0.143	0.136	0.167	0.250	0.188	0.100	0.200	0.063	0.125	0.167	0.111	0.167
	98	0.500	0.250	0.214	0.136	0.222	0.094	0.313	0.400	0.200	0.188	0.188	0.333	0.167	0.111
	100	0.333	0.438	0.286	0.455	0.333	0.375	0.250	0.250	0.300	0.375	0.250	0.167	0.389	0.167
	102		0.125	0.143	0.091	0.056	0.063		0.050		0.188	0.125	0.056	0.111	0.111
	104						0.063					0.063			
	106						0.031			0.050					
	108	0.056											0.111	0.056	
Pf-31IB1	124						0.063								
	126	0.056	0.143	0.071	0.045	0.056	0.063		0.063	0.200	0.313	0.083	0.063	0.111	0.167
	132	0.056					0.031	0.063		0.050				0.056	0.056
	134	0.056					0.031								
	139	0.333	0.429	0.214	0.273	0.444	0.406	0.313		0.250	0.063	0.167	0.375	0.278	0.389
	142							0.063	0.063	0.100	0.063				
	144		0.071	0.071											
	146	0.111	0.143				0.156		0.125	0.050	0.063	0.333	0.063	0.111	0.111
	149			0.071	0.045	0.056	0.094	0.063	0.188			0.083	0.063		
	150		0.071					0.063	0.063						
	152														0.056
	156							0.063	0.063						0.056
	162								0.063						
	164									0.100					

Locus	Allele	APS	ACS	APB	CDN	IPO	ARG	MVE	CPA	CPC	PLF	MAD	CAM	MLA	MLT
	166	0.056		0.071		0.056									
	168												0.063		
	170	0.056		0.071	0.045				0.063	0.050	0.125	0.167	0.063	0.056	0.056
	172			0.071			0.063			0.050				0.056	
	174												0.063		0.111
	176	0.056		0.071	0.045			0.188			0.125	0.083			
	178			0.071	0.136	0.111				0.050	0.063	0.083	0.063	0.056	
	180	0.167	0.071		0.045	0.056				0.100	0.063				
	182				0.045		0.063	0.063	0.063		0.125			0.056	
	184	0.056		0.143	0.182	0.111		0.125	0.250				0.188	0.167	
	186				0.091	0.111									
	188		0.071	0.071			0.031							0.056	
	190				0.045										

Table S4. Pairwise genetic differentiation. For each metric is reported the 95% confidence interval (CI) based on 10'000 bootstrap replicates. L CI_{95} = lower limit of the 95% CI; U CI_{95} = upper limit of 95% CI

		F_{ST}			G'_{ST}		
**Site 1**	**Site 2**	**Observed value**	**L CI_{95}**	**U CI_{95}**	**Observed value**	**L CI_{95}**	**U CI_{95}**
APS01	ACS02	0.016	-0.050	0.115	0.041	-0.078	0.201
APS01	APB01	0.003	-0.059	0.095	0.018	-0.101	0.187
APS01	CDN01	-0.004	-0.046	0.061	-0.004	-0.087	0.117
APS01	IPO02	0.005	-0.046	0.083	0.014	-0.081	0.145
APS01	ARG01	0.015	-0.025	0.078	0.037	-0.040	0.152
APS01	MVE01	0.005	-0.053	0.089	0.013	-0.101	0.171
APS01	CPA01	0.014	-0.030	0.082	0.035	-0.046	0.153
APS01	CPC18	-0.007	-0.048	0.053	-0.008	-0.087	0.099
APS01	PLF01	0.004	-0.046	0.079	0.010	-0.084	0.143
APS01	MAD02	0.018	-0.038	0.103	0.042	-0.061	0.183
APS01	CAM01	-0.002	-0.049	0.073	0.006	-0.080	0.133
APS01	MLA01	0.004	-0.045	0.075	0.017	-0.074	0.148
APS01	MLT01	0.029	-0.023	0.100	0.066	-0.030	0.186
ACS02	APB01	-0.030	-0.086	0.062	-0.060	-0.174	0.106
ACS02	CDN01	0.008	-0.036	0.082	0.019	-0.067	0.152
ACS02	IPO02	0.005	-0.043	0.083	0.009	-0.079	0.141
ACS02	ARG01	0.011	-0.030	0.070	0.026	-0.053	0.135
ACS02	MVE01	-0.003	-0.049	0.071	-0.009	-0.100	0.127
ACS02	CPA01	0.021	-0.037	0.110	0.047	-0.066	0.204
ACS02	CPC18	0.011	-0.038	0.087	0.028	-0.069	0.171
ACS02	PLF01	-0.010	-0.062	0.068	-0.024	-0.123	0.110
ACS02	MAD02	-0.020	-0.079	0.064	-0.044	-0.156	0.102
ACS02	CAM01	-0.011	-0.063	0.071	-0.016	-0.117	0.124
ACS02	MLA01	0.001	-0.055	0.091	0.008	-0.106	0.186
ACS02	MLT01	0.045	-0.003	0.121	0.103	0.016	0.223
APB01	CDN01	-0.018	-0.065	0.055	-0.045	-0.144	0.099
APB01	IPO02	-0.003	-0.058	0.081	-0.008	-0.120	0.151
APB01	ARG01	0.005	-0.040	0.073	0.015	-0.077	0.147
APB01	MVE01	-0.026	-0.076	0.054	-0.069	-0.183	0.090
APB01	CPA01	-0.018	-0.066	0.053	-0.042	-0.138	0.096
APB01	CPC18	-0.019	-0.068	0.057	-0.042	-0.150	0.112
APB01	PLF01	-0.016	-0.065	0.059	-0.041	-0.141	0.099
APB01	MAD02	-0.017	-0.073	0.068	-0.039	-0.156	0.118
APB01	CAM01	-0.039	-0.086	0.037	-0.086	-0.185	0.061
APB01	MLA01	-0.026	-0.077	0.052	-0.059	-0.169	0.103
APB01	MLT01	0.014	-0.040	0.093	0.038	-0.073	0.189
CDN01	IPO02	-0.026	-0.072	0.046	-0.064	-0.164	0.079
CDN01	ARG01	-0.007	-0.037	0.037	-0.016	-0.080	0.070
CDN01	MVE01	-0.003	-0.051	0.070	-0.014	-0.120	0.143
CDN01	CPA01	0.006	-0.033	0.059	0.011	-0.068	0.115
CDN01	CPC18	-0.012	-0.045	0.037	-0.030	-0.101	0.071
CDN01	PLF01	-0.020	-0.058	0.037	-0.052	-0.131	0.059
CDN01	MAD02	0.001	-0.046	0.070	-0.002	-0.103	0.135

		F_{ST}			G'_{ST}		
**Site 1**	**Site 2**	**Observed value**	**L CI$_{95}$**	**U CI$_{95}$**	**Observed value**	**L CI$_{95}$**	**U CI$_{95}$**
CDN01	CAM01	-0.018	-0.060	0.045	-0.040	-0.127	0.083
CDN01	MLA01	-0.020	-0.056	0.034	-0.048	-0.126	0.062
CDN01	MLT01	0.016	-0.029	0.080	0.036	-0.056	0.157
IPO02	ARG01	-0.002	-0.043	0.058	-0.006	-0.092	0.112
IPO02	MVE01	-0.002	-0.053	0.072	-0.016	-0.122	0.131
IPO02	CPA01	0.009	-0.035	0.073	0.016	-0.069	0.128
IPO02	CPC18	0.003	-0.041	0.066	0.006	-0.086	0.131
IPO02	PLF01	-0.008	-0.052	0.059	-0.027	-0.113	0.096
IPO02	MAD02	0.013	-0.040	0.090	0.021	-0.084	0.165
IPO02	CAM01	-0.016	-0.064	0.054	-0.036	-0.135	0.094
IPO02	MLA01	0.000	-0.052	0.076	0.000	-0.112	0.153
IPO02	MLT01	0.013	-0.038	0.085	0.027	-0.073	0.156
ARG01	MVE01	0.006	-0.034	0.063	0.012	-0.075	0.136
ARG01	CPA01	0.033	-0.004	0.086	0.077	0.002	0.175
ARG01	CPC18	-0.004	-0.037	0.042	-0.009	-0.077	0.084
ARG01	PLF01	0.003	-0.034	0.053	0.005	-0.068	0.101
ARG01	MAD02	0.010	-0.036	0.074	0.022	-0.074	0.142
ARG01	CAM01	0.002	-0.033	0.056	0.007	-0.065	0.112
ARG01	MLA01	-0.009	-0.045	0.049	-0.020	-0.097	0.102
ARG01	MLT01	0.018	-0.024	0.076	0.041	-0.044	0.154
MVE01	CPA01	0.002	-0.046	0.066	-0.001	-0.099	0.128
MVE01	CPC18	-0.002	-0.045	0.061	-0.010	-0.106	0.134
MVE01	PLF01	0.005	-0.042	0.069	0.001	-0.097	0.134
MVE01	MAD02	0.002	-0.051	0.082	-0.006	-0.116	0.151
MVE01	CAM01	-0.023	-0.065	0.042	-0.057	-0.145	0.068
MVE01	MLA01	-0.007	-0.056	0.065	-0.019	-0.127	0.131
MVE01	MLT01	0.023	-0.026	0.088	0.052	-0.049	0.178
CPA01	CPC18	0.005	-0.034	0.058	0.012	-0.066	0.120
CPA01	PLF01	-0.002	-0.044	0.056	-0.010	-0.090	0.099
CPA01	MAD02	0.001	-0.047	0.074	0.000	-0.094	0.133
CPA01	CAM01	-0.015	-0.058	0.046	-0.032	-0.118	0.086
CPA01	MLA01	0.009	-0.032	0.067	0.023	-0.061	0.137
CPA01	MLT01	0.030	-0.013	0.089	0.069	-0.016	0.179
CPC18	PLF01	-0.012	-0.051	0.047	-0.032	-0.113	0.087
CPC18	MAD02	0.003	-0.042	0.069	0.005	-0.088	0.139
CPC18	CAM01	-0.009	-0.050	0.051	-0.019	-0.105	0.107
CPC18	MLA01	-0.005	-0.048	0.061	-0.008	-0.106	0.133
CPC18	MLT01	0.003	-0.042	0.066	0.009	-0.087	0.137
PLF01	MAD02	-0.019	-0.068	0.053	-0.050	-0.150	0.086
PLF01	CAM01	-0.008	-0.056	0.063	-0.019	-0.115	0.117
PLF01	MLA01	-0.007	-0.053	0.060	-0.018	-0.112	0.116
PLF01	MLT01	0.023	-0.030	0.096	0.050	-0.054	0.183
MAD02	CAM01	-0.018	-0.069	0.065	-0.039	-0.140	0.114
MAD02	MLA01	-0.006	-0.065	0.078	-0.013	-0.133	0.153
MAD02	MLT01	0.031	-0.021	0.108	0.069	-0.036	0.210

		F_{ST}			G'_{ST}		
**Site 1**	**Site 2**	**Observed value**	**L CI$_{95}$**	**U CI$_{95}$**	**Observed value**	**L CI$_{95}$**	**U CI$_{95}$**
CAM01	MLA01	-0.027	-0.072	0.043	-0.058	-0.152	0.079
CAM01	MLT01	0.016	-0.029	0.079	0.041	-0.050	0.158
MLA01	MLT01	0.006	-0.045	0.078	0.017	-0.090	0.162
